# A Case Report of a Young Adult With Peutz-Jeghers Syndrome Presenting With Acute Small Bowel Obstruction: A Common Complication of an Uncommon Disease

**DOI:** 10.7759/cureus.65931

**Published:** 2024-08-01

**Authors:** Ashish Kuruvila, Prince Philip, Abin Thomas, Geena Benjamin

**Affiliations:** 1 Department of Radiology, Pushpagiri Institute of Medical Sciences and Research Centre, Thiruvalla, IND

**Keywords:** peutz-jeghers syndrome, small bowel obstruction, radiology, intussusception, hamartomatous polyp, mucocutaneous pigmentation

## Abstract

Peutz-Jeghers syndrome (PJS) is an autosomal dominant disorder characterized by hamartomatous polyposis of the gastrointestinal tract, melanin pigmentation of the skin and mucous membranes, and an increased risk for cancer. Radiological imaging, contrast studies, and scopy-directed biopsies confirm the diagnosis and help in surveillance. Hamartomatous mucosal polyps, which are characterized by a central core of branching smooth muscle connected to a mucosa unique to the site of origin, are pathognomonic for PJS. We present the case of a young male with a history of pain in the abdomen and vomiting. The patient had mucocutaneous pigmentations on the buccal mucosa. CT scan revealed jejuno-jejunal intussusception with multiple small and large bowel polyps causing acute intestinal obstruction. Intraoperatively, jejunal polyps were found to be the cause of jejuno-jejunal intussusception. Histopathology revealed hamartomatous polyps of PJS. Our interest in this case is due to the uncommon case of intussusception in an adult where radiological imaging played an important role in diagnosis.

## Introduction

Dr. Jan Peutz first identified Peutz-Jeghers syndrome (PJS) as a syndrome in 1921. This is an autosomal dominant polypoid syndrome, presenting with gastrointestinal hamartomatous polyps and mucocutaneous pigmentation. Histopathology and clinical symptoms are used to confirm the diagnosis. With 64% of cases occurring in the small bowel, stomach, and colon, hamartomatous polyps are most frequently identified in these three organs [1].

Dark blue to brown macules can be found in the pigmentations surrounding the lips, eyes, nose, buccal mucosa, palmar surfaces of the hands, genitalia, and perianal region. To the uninformed eye, these pigmentations may look like freckles, but the involvement of the buccal mucosa rules out that possibility. Intussusceptions can recur due to the polyps acting as a trigger point, requiring immediate, potentially fatal surgery. The primary cause for concern is from the heightened likelihood of many cancers due to the disease's molecular etiology [2].

## Case presentation

**Figure 1:**
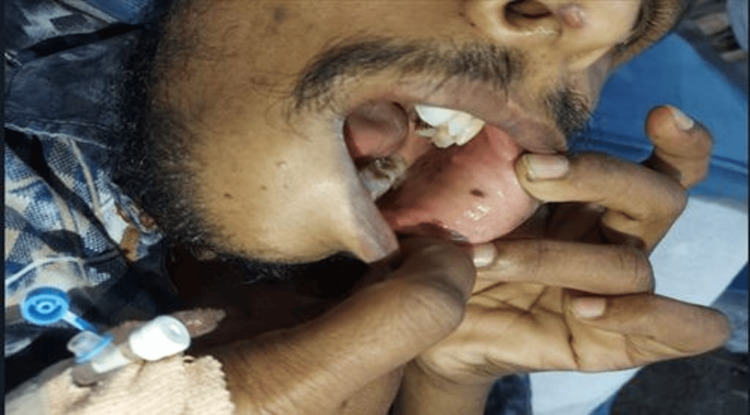
Hyperpigmented macules on the patient's buccal mucosa

A 30-year-old male presented to the emergency department with complaints of acute-onset abdominal pain and vomiting for four days. It was not associated with any fever, hematemesis, or bloody diarrhea. The patient gave a family history of colonic polyps in his father and paternal grandfather. One of the patient's relatives had expired due to a colorectal malignancy. On palpation, pain and tenderness were felt over the left hypochondrial and umbilical regions. Hyperpigmented macular lesions were seen over the buccal mucosa (Figure 1); however, no similar lesions were seen in the nose, axilla, hands, feet, or genitalia. Blood investigations were advised which revealed leukocytosis (white blood cell 18,000/mm^3^). Other blood tests were within normal limits.

**Figure 2:**
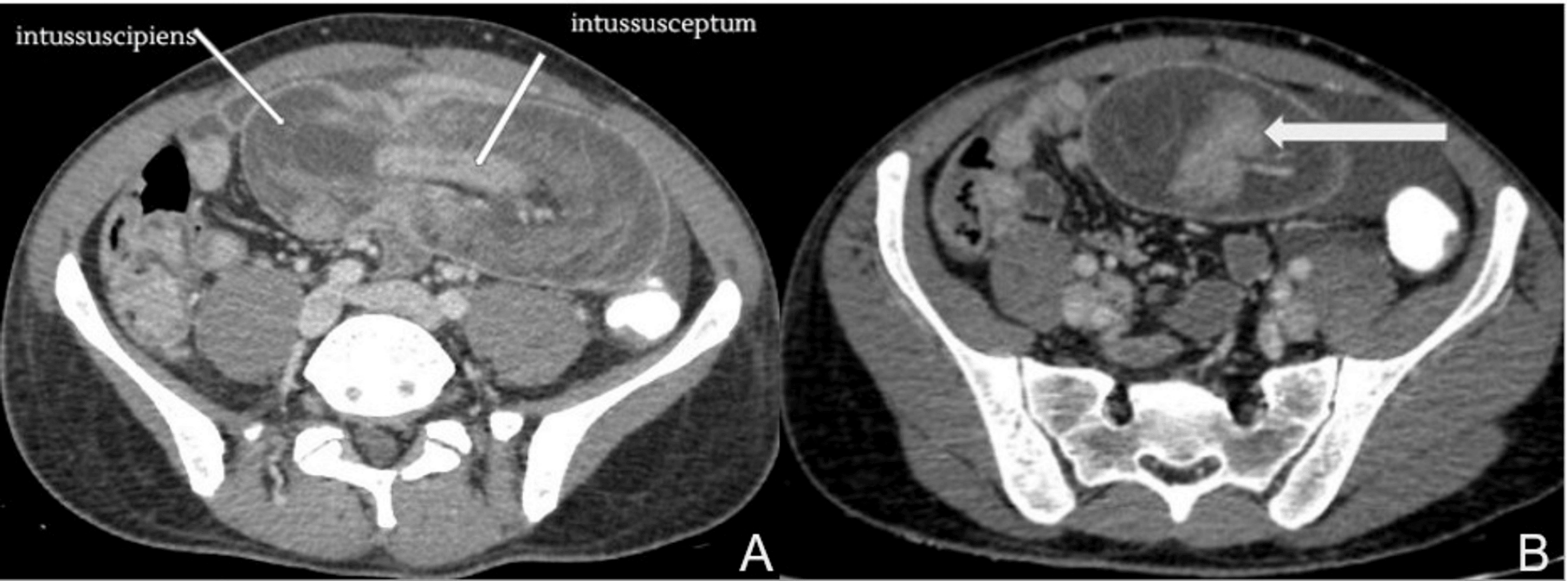
CT scan of the abdomen and pelvis with rectal and intravenous contrast on axial plane in venous phase showing (A) a bowel-within-bowel appearance involving the long segment of the small bowel (jejuno-jejunal), suggestive of intussusception with thickened and edematous wall of intussuscipiens, and (B) the largest polyp seen in the inferiormost aspect of intussusception acting as lead point (thick white arrow)

**Figure 3:**
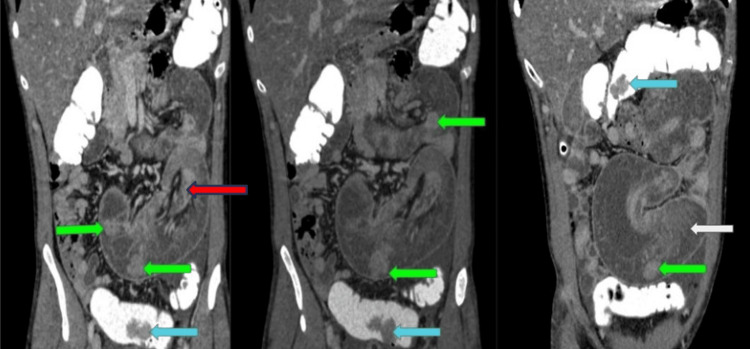
Serial coronal images of CT of the abdomen and pelvis with intravenous and rectal contrast showing mildly dilated jejunal loop (white arrow) with telescoping of jejuno-jejunal intussusception (red arrow) along with small bowel (green arrow) and large bowel (blue arrow) polyps

CT scan of the abdomen and pelvis with intravenous and rectal contrast was performed which revealed jejuno-jejunal intussusception containing several polyps (the largest measured 32 mm) with mildly dilated proximal jejunum (Figure 2). Several other polyps were also observed in the duodenum, jejunum, and ascending, transverse, and sigmoid colon (Figure 3). Based on the patient's family history along with the presence of mucocutaneous hyperpigmentation and multiple polyps in the gastrointestinal tract, the diagnosis of PJS with jejuno-jejunal intussusception causing acute intestinal obstruction was considered.

**Figure 4:**
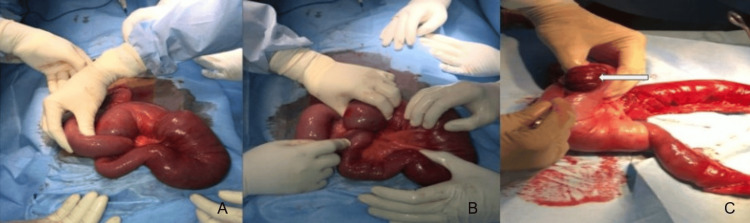
Operative images (A and B) showing jejuno-jejunal intussusception with polyp inside and (C) polyp causing intussusception (white arrow)

Given the patient's imaging findings, an emergency exploratory laparotomy was done. Operatively, jejuno-jejunal intussusception of the proximal jejunum was seen approximately for a length of 60 cm. The neck of the intussusception was about 15 cm from the duodeno-jejunal flexure (Figure 4). Edema and ischemic changes of the intussuscipiens with congested mesentery along with polyps were observed in the small and large bowel. The mid-transverse colon and the sigmoid colon each had a polyp measuring 2×2 cm and 1.5×2 cm, respectively. Hence, resection of the intussuscepted segment and jejuno-jejunal anastomosis along with the resection of colonic polyps was performed for definitive management.

**Figure 5:**
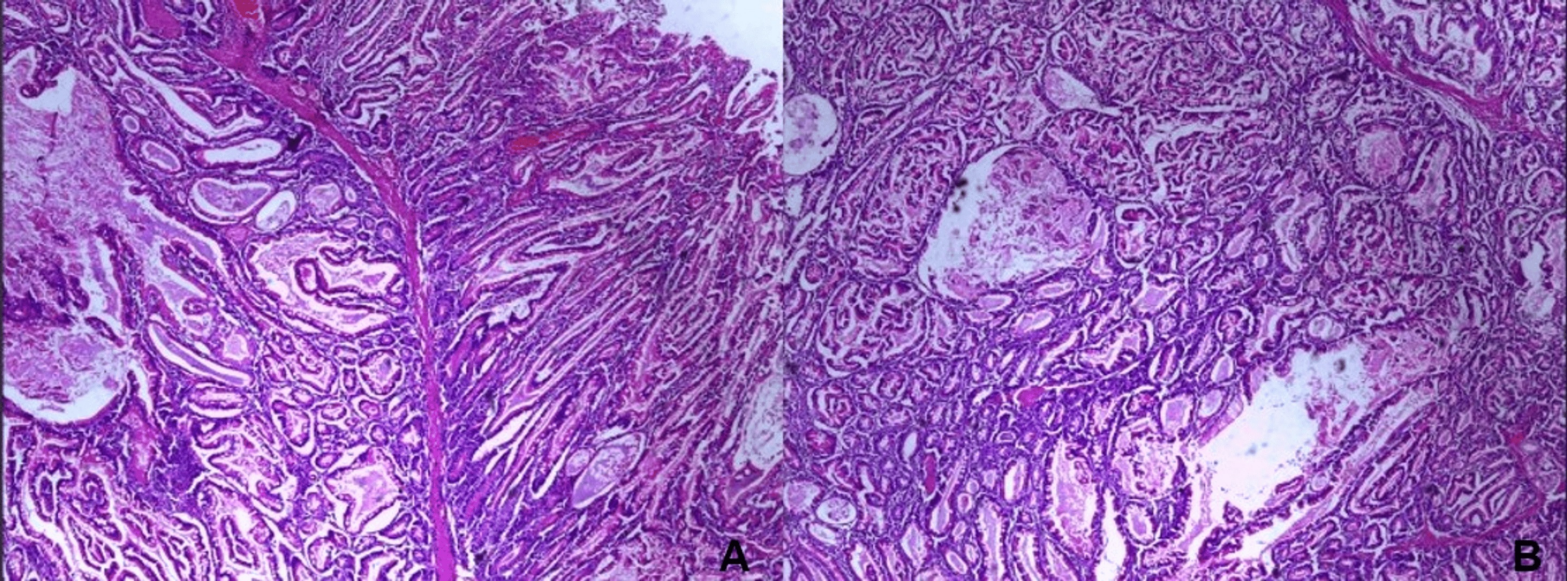
(A and B) Photomicrograph (hematoxylin-eosin stain) demonstrating cells arranged in villoglandular architecture with central intervening arborizing smooth muscle cores, consistent with Peutz-Jeghers polyp

On histopathology, the jejunum showed multiple hamartomatous polyps (7) which was consistent with the diagnosis of PJS (Figure 5). The largest jejunal polyp specimen measured 4.5×3.5×2.5 cm. No signs of malignant transformation were detected. The intussusception with the largest polyp was the leading point, and all the lymph nodes showed reactive changes only. The postoperative period was uneventful, and the patient is currently on regular follow-up as recommended.

## Discussion

We present a case of PJS involving multiple hamartomatous polyps primarily located in the jejunum of a 30-year-old male patient who had acute small intestinal obstruction due to jejuno-jejunal intussusception. The patient exhibited every hallmark indication of PJS, including multiple gastrointestinal polyps, mucocutaneous pigmentation, and a positive family history.

In children, intussusception is a rather common emergency condition that is typically idiopathic [3]; however, hypertrophied Peyer's patches secondary to viral intestinal infections have also been proposed. On the other hand, adult intussusception is rare, with tumors accounting for 70-90% of diagnoses [4]. Roughly around half of them are cancerous and the other half are benign.

Intussusception occurs when a bowel loop, known as "the intussusceptum" in its mesenteric region, telescopes into the lumen of an adjacent segment, known as "the intussuscipiens." This phenomenon puts the mesenteric vascular flow of this portion of the bowel at risk, hindering the passage of intestinal content and impairing peristalsis, and may even cause intestinal obstruction [5]. Intussusception is a rare cause of intestinal obstruction in adults, with a reported incidence of 1-5% [6].

With an estimated prevalence of one in 100,000 people, PJS is an uncommon condition [7]. Multiple neoplasms, mucocutaneous hyperpigmentation, and gastrointestinal hamartomatous polyps are its characteristic features. It is caused by autosomal dominant inheritance. There is no racial predominance, and this entity can be observed in patients who are male or female. The World Health Organization (2010) established the following diagnostic standards for PJS: any number of Peutz-Jeghers polyps in individuals with a family history of the condition, three or more Peutz-Jeghers polyps that have been histologically confirmed, distinctive prominent mucocutaneous pigmentation combined with a family history of PJS, or any combination of Peutz-Jeghers polyps and distinctive prominent mucocutaneous pigmentation [2]. Our subject of interest fulfilled all the abovementioned criteria.

Adult cases of acute intestinal intussusception are uncommon. It occurs for only 1-5% of intestinal obstruction in adults and constitutes 5% of all intussusceptions [8]. Adult intussusception is uncommonly caused by hamartomatous polyposis. However, among patients with PJS, this consequence is the most frequent presentation. As PJS is a rare condition, patients frequently go undiagnosed for many years unless they present with intussusception as in our case.

A germline mutation in STK11, which has been connected to an increased risk of breast, gastrointestinal, and gynecologic malignancies, is inherited by patients with PJS [9]. The final diagnosis of multiple hamartomatous polyps of the PJS type is determined by histopathology. Genetic analysis has been used to diagnose PJS by looking for mutations and loss of heterozygosity at the 19p13.3 locus of the PJS susceptibility gene, STK11/LB1 [10]. It affects half the people who have sporadic and familial PJS. Up to 45% of people have a negative family history, which could be the result of a de novo germline mutation [11]. The mutation in these patients also increases their likelihood of developing neoplasia since this gene functions as a tumor suppressor [12]. The fact that gastrointestinal cancers develop apart from the hamartomatous polyps suggests that they are not preneoplastic precursor lesions. 

PJS-type hamartomatous polyps have a distinctive histology characterized by smooth muscle growth that extends into the lamina propria and forms a pattern resembling arborization. Polyps that are hamartomatous are typically regarded as benign [13]. However, there is an increased risk of cancer in the stomach and other extraintestinal areas when it is linked to PJS [14]. According to Hearle et al., by the age of 70 years, patients with PJS had an 85% chance of becoming malignant, a fourfold increased risk over the general population [15].

Various imaging studies are carried out that aid in the management of PJS. These consist of colonoscopy, intraoperative enteroscopy, endoscopic ultrasound, double-balloon enteroscopy, CT enterography, MRI enteroclysis, capsule endoscopy, and CT scan with oral contrast agent. These patients need to be followed up every two years for upper gastrointestinal endoscopy and small bowel radiography as well as three yearly colonoscopy, ultrasound, carcinoembryonic antigen (CEA), and PAP smear [16]. Treatment options for patients with PJS are endoscopic removal of all polyps or, in case of intussusception, a laparotomy with intraoperative enteroscopy.

Imaging is done in PJS for the periodic surveillance, diagnosis, and identification of problems [17]. With a diagnostic accuracy of roughly 83% in cases of intussusception linked to PJS, the majority of research suggests that the CT is the most reliable preoperative diagnostic tool [18] which clarifies our strategy in managing our patient's diagnosis. Patients with PJS are more likely to experience a range of problems and tumors, especially breast and gastrointestinal cancers [19].

Therefore, in order to prevent problems and enhance patient outcomes, patients need to be routinely monitored. The goal of periodic observation and the excision of bigger polyps is to lower the risk of problems in PJS. Therefore, patients should have a yearly complete blood cell count along with an annual physical examination that includes a check of the belly, testicles, breasts, and pelvis. Also, magnetic resonance cholangiopancreatography (MRCP) is recommended for increased risk of biliary cancers, per the American College of Gastroenterology Clinical Guidelines [20].

## Conclusions

PJS is an uncommon hereditary condition that calls for specialized care and an interdisciplinary approach. Some patients may present with acute complications such as intussusception for the first time. Adult intussusception patients typically have nonspecific, persistent symptoms, which delay diagnosis. Early diagnosis is critical because gastrointestinal tract cancers should be detected early and surveillance should be made available to afflicted patients and their at-risk family members from a young age.
